# Web-based and mixed-mode cognitive large-scale assessments in higher education: An evaluation of selection bias, measurement bias, and prediction bias

**DOI:** 10.3758/s13428-020-01480-7

**Published:** 2020-10-01

**Authors:** Sabine Zinn, Uta Landrock, Timo Gnambs

**Affiliations:** 1 German Institute for Economic Research, Berlin, Germany; 2 Leibniz Institute for Educational Trajectories, Bamberg, Germany; 3 Johannes Kepler University Linz, Linz, Austria

**Keywords:** Mode effect, Web-based testing, Computerized testing, Measurement invariance, Selection effect, Higher education

## Abstract

Educational large-scale studies typically adopt highly standardized settings to collect cognitive data on large samples of respondents. Increasing costs alongside dwindling response rates in these studies necessitate exploring alternative assessment strategies such as unsupervised web-based testing. Before respective assessment modes can be implemented on a broad scale, their impact on cognitive measurements needs to be quantified. Therefore, an experimental study on *N* = 17,473 university students from the German *National Educational Panel Study* has been conducted. Respondents were randomly assigned to a supervised paper-based, a supervised computerized, and an unsupervised web-based mode to work on a test of scientific literacy. Mode-specific effects on selection bias, measurement bias, and predictive bias were examined. The results showed a higher response rate in web-based testing as compared to the supervised modes, without introducing a pronounced mode-specific selection bias. Analyses of differential test functioning showed systematically larger test scores in paper-based testing, particularly among low to medium ability respondents. Prediction bias for web-based testing was observed for one out of four criteria on study-related success factors. Overall, the results indicate that unsupervised web-based testing is not strictly equivalent to other assessment modes. However, the respective bias introduced by web-based testing was generally small. Thus, unsupervised web-based assessments seem to be a feasible option in cognitive large-scale studies in higher education.

**Electronic supplementary material:**

The online version of this article (10.3758/s13428-020-01480-7) contains supplementary material, which is available to authorized users.

Large-scale educational studies collect information on individuals’ domain-specific competencies and general cognitive abilities to study their relevance for educational choices and peoples’ successful participation in society (see Blossfeld, Maurice and Schneider, 2019; Reiss, Obersteiner, Heinze, Itzlinger-Bruneforth and Lin, 2019; Strietholt and Scherer, 2018). For example, the *Programme for International Student Assessment* (PISA; http://www.oecd.org/pisa/) and the *Programme for the International Assessment of Adult Competencies* (PIAAC; https://www.oecd.org/skills/piaac/) assess the competence levels of adolescents and adults from over 40 countries around the world in, among others, reading, mathematics, and science. Similarly, the German *National Educational Panel Study* (NEPS; https://neps-data.de) examines the development of domain-specific competencies from birth to adulthood along different stages of the life course in large and nationally representative samples. Each of these studies strives to collect cognitive data that is comparable across respondents and allows unbiased conclusions on pertinent research questions. Therefore, educational large-scale assessments typically adopt supervised and highly standardized test settings: all respondents receive the same test under identical (or highly similar) conditions such as in a classroom at the students’ schools or the respondents’ private homes, while being continuously monitored by a trained test administrator. A major obstacle for these types of assessments is their costs in terms of money, administrative burden, and personal resources which can make their implementation in large-scale studies (with thousands of participants) prohibitive. Limited time resources of participants (e.g., of full-time employees) or respondents who move frequently or travel a lot (e.g., university students) can further endanger response rates in these studies because timely appointments for supervised testing cannot be reached (e.g., Haunberger, 2011; Kuhnimhof, Chlond and Zumkeller, 2006). To mitigate these challenges, web-based settings or mixed-mode designs adopting different data collection modes for different respondents have been considered (Al Baghal, 2019). To what degree measurements obtained in these designs might be affected by the change in assessment settings is an ongoing question (for reviews see Delgado, Vargas, Ackerman and Salmerón, 2018; Steger, Schroeders and Gnambs, 2020; Wang, Jiao, Young, Brooks and Olson, 2007, Wang, Jiao, Young, Brooks and Olson, 2008). Unfortunately, most previous research on this issue relied on small and highly selective, *adhoc* recruited non-probability samples which make it difficult to draw generalizable conclusions. Some authors (Schroeders and Wilhelm, 2011) even argue that mode equivalence depends on the specific construct and the studied population and that the testing program necessitates highly targeted equivalence research. Particularly, for educational large-scale assessments, this requirement is not yet fully met. The present research addresses this shortcoming and reports on a feasibility study that implemented a web-based cognitive test component in an ongoing large-scale assessment. The research evaluates to what degree cognitive test scores from unsupervised web-based testing are comparable to supervised assessments. In contrast to previous research (e.g., Al Baghal, 2019; Gooch, 2015; Schroeders & Wilhelm, 2011), a multi-perspective approach is adopted to study whether mode differences introduce selection bias, measurement bias, or prediction bias.

## Mixed blessings of web-based cognitive testing

For large-scale cognitive assessments^1^, unsupervised web-based tests might tackle the problem of increasing financial costs and low participation probabilities (Gnambs, Batinic and Hertel, 2011). Higher response rates can be expected for groups that travel a lot or have limited time resources because in web-based assessments the timing and location of the test can be chosen freely. Moreover, no additional costs arise for renting test centers, instructing and paying test administrators, printing test material, or buying and maintaining software and hardware for computerized assessments. Finally, construct-irrelevant influences such as test anxiety might reduce in the absence of a supervising authority (Stowell and Bennett, 2010). On the downside, unsupervised web-based testing might hold several pitfalls (see Kroehne, Gnambs and Goldhammer, 2019): in an unsupervised setting test-takers might cheat (e.g., searching answers on the Internet) and behave dishonestly (e.g., letting someone else answer the test). Indeed, a meta-analysis (Steger et al., 2020) suggests that respondents cheat in unsupervised web-based testing if they have the opportunity to do so, even if the outcome of the assessment does not yield personal consequences. Moreover, various disturbances such as background noise or other people being able to see the test taker’s responses can potentially further influence the test-taking behavior (see Gnambs and Kaspar, 2015, for respective evidence in the context of survey research). Finally, technological differences such as different screen sizes or input devices (e.g., mouse versus touchscreen) might introduce further construct-irrelevant variance that could distort measurements in unsupervised web-based settings, particularly when the assessment can be accessed using mobile and non-mobile devices (Brown and Grossenbacher, 2017). All this taken together limits the comparability of test scores. In large-scale studies with mixed-mode designs, the benefits of implementing unsupervised web-based testing will only outweigh its drawbacks, if the observed test scores are comparable to those obtained under supervised conditions.

## Mode effects for web-based cognitive testing

Test score equivalence is given if the rank orders of individuals’ test scores do not change depending on the testing mode and the test score distributions are comparable under different assessment conditions (AERA, APA,, & NCME, 2014). Test score equivalence can be studied in experimental designs by randomly assigning individuals to different modes and administering identical cognitive tests in each mode.^2^ Only if the individuals assigned to the different modes are similar concerning important background characteristics and, thus, are comparable between the experimental groups the psychometric properties of the administered measure can be evaluated to corroborate equivalence between assessment conditions. If similar measurement models can be corroborated, predictive invariance might be studied to evaluate whether the cognitive scores predict important outcomes comparable in the different modes. Predicting later life outcomes such as occupation based on cognitive measures is of particular interest in educational science where the attempt is made to relate both (e.g., Blossfeld, Schneider and Doll, 2009).

## Mode effects and selection bias

Even if individuals are assigned at random to different testing modes, participation probabilities can depend on different respondent characteristics and these dependencies might differ between modes. Thus, selection bias is likely to occur in the statistical analyses of test score equivalence (Keiding and Louis, 2018). For example, in a study among first-year college students in the United States Sax, Gilmartin and Bryant (2003) found a lower propensity to participate in a web survey (as compared to an identical paper-based survey) for women and a higher participation propensity for students attending a college far away from home. Similarly, psychological characteristics such as respondents’ intrinsic motivations and trust in anonymity seem to influence repeated participation in longitudinal web-based studies (Stiglbauer, Gnambs and Gamsjäger, 2011). Also, differences in people’s Internet access conditions and their Internet usage behavior might affect their participation propensities in web-based assessments whereas they unlikely do so for paper-based formats (e.g., Fan and Yan, 2010). Thus, different respondent characteristics can govern the decision of whether to participate in an unsupervised or a supervised test. The situation is even worse if respondents can choose their preferred testing mode or nonresponders in a supervised assessment are switched to the unsupervised web-based mode. In such situations, the comparability of the observed samples for the different mode groups is no longer guaranteed because people select themselves into certain modes with unequal probabilities (e.g., Schouten, van den Brakel, Buelens, van der Laan and Klausch, 2013). So far, mode-specific selection effects have not yet been examined for web-based cognitive assessments.

## Mode-effects and measurement bias

A test can provide systematically different information about the construct to be measured under different testing modes. If individuals who have identical values on the latent construct (e.g., mathematical competence) exhibit different probabilities of obtaining the same observed score depending on their test group membership (e.g., web-based versus paper-based assessment) the test exhibits measurement bias (AERA, APA,, & NCME, 2014). This typically implies different factor structures in the subgroups and, thus, a lack of measurement invariance (cf. Schroeders and Gnambs, 2020). Research on measurement mode effects for supervised paper-based and computerized cognitive tests has a long tradition (for reviews and meta-analyses see Mead and Drasgow, 1993; Wang et al., 2007, 2008). In general, the meta-analyses point to test score equivalence across test media for general ability tests and domain-specific competence tests. Thus, whether a test is presented on paper or computer makes little difference for the measured construct. However, several exceptions highlight that invariance across test media depends on the specific measure in question and on the study population (e.g., Lenhard, Schroeders and Lenhard, 2017; Robitzsch et al., 2017). For example, Lenhard et al. (2017) showed that school children worked faster on a reading comprehension test when presented on screen as compared to paper and, at the same time, produced more errors. Similarly, in PISA measurement mode effects were observed for mathematics, science, and reading tests after switching from paper-based competence testing to supervised computerized forms (Robitzsch et al., 2017). These tests were more difficult when presented on a computer (as compared to paper). Nevertheless, overall the transition from paper to computer does not seem to lead to pronounced changes in test results (e.g., Kroehne, Buerger et al., 2019; Schroeders & Wilhelm, 2011).

In contrast, empirical studies on the comparability of supervised paper-based and computerized cognitive assessments with unsupervised web-based ones typically show inflation of the test scores in unsupervised web-based studies. Meta-analytic evidence (Steger et al., 2020) showed score differences of about Cohen’s *d* = 0.20 in favor of unsupervised cognitive testing. Despite the low-stake settings in unsupervised web-based studies that yielded no individual consequences for the test takers contingent on their test performance, cheating (i.e., searching the correct answers on the Internet) might have distorted the web-based assessments. A limitation of most of the available studies is that they were based on *adhoc* recruited student samples without accounting for potential sample selection effects. Also, frequently setting effects (supervised versus unsupervised) and test media effects (paper versus computer) were confounded making it difficult to draw clear conclusions. Only recently, Al Baghal (2019) used the *United Kingdom Household Longitudinal Study* (UKHLS) to compare reasoning and working memory test scores between a supervised computerized cognitive assessment and an unsupervised web-based one. Similar to Steger et al. (2020), he found that the test scores obtained in the presence of a test administrator were significantly lower than those obtained in the unsupervised web-based setting. Although Al Baghal (2019) considered self-selection processes into particular modes, the mode-specific differences in the test scores remained. Thus, setting effects seem to have contributed to these differences. So far, there is no evidence whether these findings generalize to different cognitive domains and populations. Thus, it is essential to explore potential media effects on cognitive assessments for each specific test instantiation before addressing substantive research questions with cognitive data from mixed-mode designs.

## Mode effects and predictive bias

Assessment modes contribute to a predictive bias if the prediction of a criterion based on a test varies with group membership (Millsap, 2007). If important outcomes, for example, job success (Gnambs, 2017) or psychological health (Wraw, Deary, Der and Gale, 2016) exhibit different associations with cognitive test scores depending on whether they were obtained in an unsupervised web-based assessment or a supervised context, mode effects result in differential predictions. So far, predictive bias has been primarily studied in the employment context to evaluate whether cognitive ability tests are biased, for example, against a specific gender or certain ethnic groups (see Berry, 2015). In contrast, mode effect research has been surprisingly silent on this topic. Beaty et al. (2011) evaluated the predictive validities of various non-cognitive measures (e.g., a conscientiousness scale) for selecting job candidates. Their analyses detected only negligible mode effects suggesting equivalent predictions for unsupervised and supervised settings. Whether these results can be generalized to cognitive measures administered in low-stake test settings is an open question.

## Current study

**Fig. 1:**
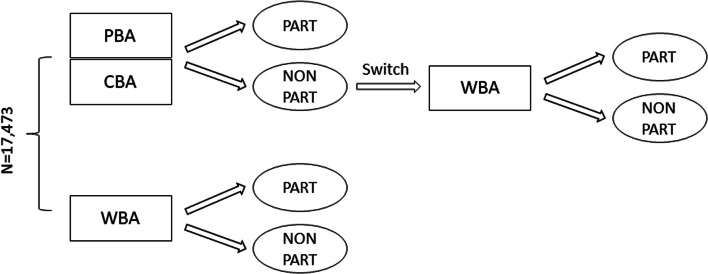
Mixed-mode design of the competence assessment with PBA = standardized and supervised paper-based assessment, CBA = standardized and supervised computer-based assessment, WBA = unstandardized and unsupervised web-based assessment, PART = participation, and NONPART = nonparticipation

As of yet, no study has comprehensively investigated the selection bias, measurement bias, and predictive bias introduced by unsupervised web-based cognitive testing in educational large-scale assessments. This study addresses this shortcoming by examining potential mode effects for the measurement of scientific literacy (i.e., the knowledge of basic scientific facts and the understanding of scientific processes; see Hahn et al., 2013) among university students taking part in an ongoing German large-scale assessment. A mode experiment was established that randomly assigned students to supervised paper-based testing, supervised computerized testing, or unsupervised web-based testing. Students that refused to take part in the supervised setting were subsequently asked to switch to the unsupervised web-based mode. This procedure resulted in a complex mixed-mode design (see Fig. 1) that allowed us to examine mode effects (paper versus computer), setting effects (supervised versus unsupervised) as well as self-selection effects (random assignment versus mode-specific nonresponse). Importantly, the analyses will disentangle selection effects arising from nonrandom mode-specific nonresponse from mode-specific differences in measurement properties of the administered instruments and mode-specific distortions in outcome predictions. This gives the unique opportunity to examine different types of mode effects. Our research is guided by three research questions (*RQ*):

*RQ1: Does unsupervised web-based testing affect students’ nonresponse propensities differently than supervised testing with regard to individual characteristics and learning environment?*

Meta-analyses on nonresponse rates for distinct survey modes have consistently shown substantially lower participation rates in unsupervised web-based research as compared to interviewer-led surveys (e.g., Daikeler, Bošnjak and Manfreda, 2020; Weigold, Weigold and Natera, 2018). However, it is still not entirely clear whether mode-specific participation rates are also associated with relevant background characteristics of the test takers and, thus, lead to nonrandom samples. Therefore, we evaluated whether (a) sociodemographic characteristics (e.g., gender, parenthood), (b) psychological traits (competences, personality), or (c) university and study-related characteristics (university type, study enjoyment) differently influenced the willingness of students to take part in the testing depending on the assessment mode.

*RQ2: Does unsupervised web-based testing affect the measurement of the latent constructs by violating the assumption of measurement invariance?*

Although previous research does not suggest testing mode effects with regard to a computerized as compared to paper-based test presentation (e.g., Mead & Drasgow, 1993; Wang et al., 2007, 2008), severe test score inflation has been observed for unsupervised web-based testing (Steger et al., 2020). However, most of these analyses did not account for potential confounding by mode-specific selection bias. In our analysis, on the other hand, we evaluated whether these results can be corroborated even after controlling for nonrandom participation probabilities.

*RQ3: Does unsupervised web-based testing affect the longitudinal prediction of relevant criteria?*

In applied settings, the differential validity of psychological instruments with regard to different respondent characteristics has been routinely scrutinized (see Berry, 2015). To what degree different assessment modes might also contribute to a prediction bias has, so far, been largely neglected, particularly for cognitive measures obtained in large-scale studies. Therefore, we evaluated whether mode effects distorted the longitudinal prediction of students’ performance, academic self-concept, study-related helplessness, and intention to quit the study program about six months after the cognitive assessment.

## Method

### Sample and Procedure

The participants were part of the longitudinal NEPS (Blossfeld, Roßbach and von Maurice, 2011) that follows representative samples of German children, adolescents, and adults across their life courses. The present study focuses on the fifth wave of the NEPS examining a sample of *N* = 17,473 (61% female) students in their third university year. Their mean age was 24.3 years (SD = 3.8). The students attended different institutions of higher education and study programs. Twenty-four percent of them were enrolled in universities of applied sciences, whereas the rest went to general universities. Most respondents studied humanities or cultural studies, only about 22% of them were enrolled in natural science programs. The analyses of predictive bias (*RQ3*) were limited to a subsample of *n* = 1825 students who were enrolled in natural science courses because for these students’ more pronounced associations between scientific literacy and the studied outcomes (see below) was expected.

The sample was randomly assigned to a supervised or unsupervised assessment mode (see Fig. 1). A total of *n* = 5371 students was asked to complete supervised, paper-based tests (PBA)^3^ and *n* = 3431 students were assigned supervised computerized tests (CBA) that were presented on bring-in notebooks. The tests were administered in small groups in dedicated rooms at the students’ universities. All test administrators received a 2-day training to ensure comparable and highly standardized administration conditions. A third group of *n* = 8671 students received unsupervised web-based tests (WBA) that had to be finished on their private notebooks or personal computers^4^. Participants were invited by email (and reminded twice) to complete the test on their home computers. Finally, students originally assigned to the supervised PBA or CBA conditions but refusing participation (i.e., nonresponders; *n* = 6804) were subsequently invited to complete the web-based test (WBA-switch).

All participants received an incentive of 20 euros. The size of the incentive was the same for all students regardless of the assessment mode, their courses, or the study subjects. Further details on the experimental procedure and the fieldwork are given in Prussog-Wagner, Weiß, Aust and Weber (2013).

### Measures

**Fig. 2:**
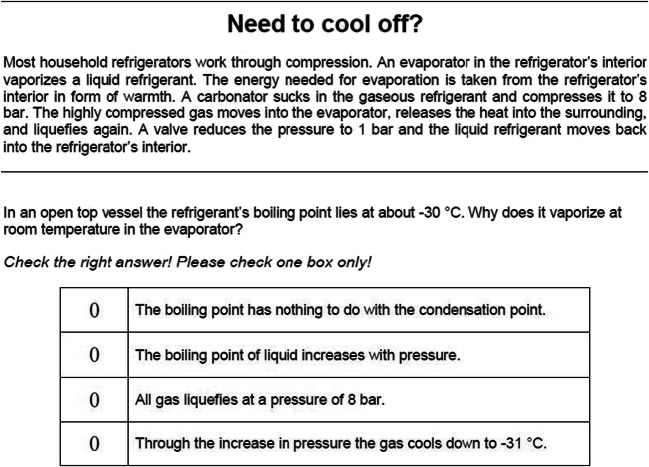
Example item of the scientific competence test administered in the NEPS. Copyright Leibniz Institute for Educational Trajectories (LIfBi). Reproduced with permission

Scientific literacy was measured with 29 items that were specifically constructed for administration in the NEPS. Of these, 16 items were dichotomous and 19 items polytomous. Similar to PISA (OECD, 2006), the test measured scientific knowledge of basic scientific concepts and facts as well as the understanding of scientific processes in the area of health, environment, and technology (see Hahn et al., 2013). An example item is given in Fig. 2. The testing time was limited to a maximum of 29 minutes. Following the psychometric model established for the NEPS (Pohl and Carstensen, 2013), each of the dichotomous items was scored with one point, whereas each of the polytomous items received half a point for each response category. In total, respondents could achieve between 0 and 36 points. The responses were scaled using a unidimensional one-parametric item response model (see Pohl & Carstensen, 2013). Respondent proficiencies were derived as weighted maximum likelihood estimates (Warm, 1989).

#### Predictors of nonresponse

A total of 28 variables were used to model mode-specific nonresponse. These included sociodemographic information (e.g., gender, year of birth), student and university characteristics (e.g., type of study and university), achievement indicators (e.g., mathematical competence, grades), personality (e.g., self-esteem), and previous participation behavior in the NEPS. A detailed description of all variables including summary statistics is given in the supplement material. The large number of predictor variables aims at predicting mode-specific response probabilities as accurately as possible. Only well-performing nonresponse models allow deriving propensity scores that properly compensate for selection bias arising due to nonrandom nonresponse (Rosenbaum and Rubin, 1985; Rubin, 1997).

#### Criterion variables

For the analysis of prediction bias, four criterion variables were selected that were collected in the sixth wave of the NEPS about six months after the competence assessment. First, the self-reported grade point average was measured with a single item inquiring about the average grade for the academic achievements to date in the current study program. Responses were given in an open response field with valid values ranging from 1 (= best grade) to 5 (= failing grade). For the analyses, the responses were reverse coded to reflect a better achievement at higher values (*M* = 2.30, *SD* = 0.55). Second, the academic self-concept was measured with four items from Dickhäuser, Schöne, Spinath and Stiensmeier-Pelster (2002) on seven-point response scales from 1 “low” to 7 “high” (*M* = 4.91, SD = 0.90). The omega reliability was good with ω = .86. Third, study-related helplessness was assessed with three items (see Jerusalem and Schwarzer, 1993) on five-point response scales from 1 “does not apply at all” to 5 “applies completely” (*M* = 1.99, *SD* = 0.86). The scale resulted in a reliability of ω = .87. Finally, students’ intention to quit the study program was measured with five items from Trautwein et al. (2007) on four-point response scales from 1 “does not apply at all” to 4 “applies completely” (*M* = 1.46, *SD* = 0.55). The reliability was good with ω = .86.

### Statistical analyses

#### Analyses of self-selection bias

We studied self-selection into the three modes PBA, CBA, and WBA^5^ by estimating a logit model with participation (0 = nonresponse, 1 = participation) as the dependent variable. Interaction effects between the previously described predictors^6^ and the modes constituted the independent variables of the model. That way, we could examine mode-specific self-selection effects. Missing values in the predictors (see supplement material for respective descriptive information) were imputed 20 times by chained equations (van Buuren and Groothuis-Oudshoorn, 2010) using sequential regression trees for continuous variables (Burgette and Reiter, 2010) and polytomous regression models for categorical variables (White, Daniel and Royston, 2010).

#### Correction for selection effects

To derive propensity scores compensating for mode-specific nonrandom unit nonresponse, we estimated four single nonresponse models, one for each mode (i.e., PBA, CBA, WBA, and WBA-switch). We applied logit models for this purpose using all the predictors described before as independent variables and participation (0 = nonresponse, 1 = participation) as the dependent variable. Based on these models and conditioned on the predictors with a significant (*p* < .05) effect, participation probabilities *p*_*im*_ were predicted for each student *i* in each mode *m*. Then, the related mode-specific propensity scores *w*_*im*_ were derived as

$$ {w}_{im}=\left\{\begin{array}{c}{p}_{\mathrm{i}1}^{-1},\kern0.5em m=\mathrm{PBA},\\ {}{p}_{\mathrm{i}2}^{-1},\kern0.5em m=\mathrm{CBA},\\ {}{p}_{\mathrm{i}3}^{-1},\kern0.5em m=\mathrm{WBA}\\ {}{\left(1-{p}_{\mathrm{i}1}\right)}^{-1}{p}_{\mathrm{i}4}^{-1},\kern0.5em m=\mathrm{WBA}-\mathrm{switch}\ \mathrm{from}\ \mathrm{PBA}\\ {}{\left(1-{p}_{\mathrm{i}2}\right)}^{-1}{p}_{\mathrm{i}4}^{-1},\kern0.5em m=\mathrm{WBA}-\mathrm{switch}\ \mathrm{from}\ \mathrm{CBA}\end{array}\right.. $$

Note that each propensity score maps the inverse participation probability of a student in the specific mode, in the cases of WBA-switch additionally conditioned on the nonparticipation in the previous modes (PBA and CBA). In subsequent analyses, these propensity scores were used as weighting factors.

#### Analyses of measurement bias

Measurement bias was analyzed by examining differential test functioning (DTF). A test indicates DTF if the relationship between the measured latent proficiency and the expected test scores differs between groups, although the true differences on the latent variable are held constant (Raju, van der Linden and Fleer, 1995). Thus, DTF examines how item bias accumulates to produce biased test scores for the comparison of groups. To this end, we fitted a one-parametric item response model (Rasch, 1960) to the test responses for the different assessment modes (PBA, CBA, WBA, WBA-switch) while accounting for the non-random nonresponse (see above), yielding an individual test score function for each mode. The fit of the measurement model for each mode was evaluated using a weighted mean square statistic for each item (Linacre, 2003) that quantifies the discrepancy between the observed and model-implied item responses. In line with prevalent recommendations, values of these statistics below 1.20 were considered acceptable (cf. Smith, Rush, Fallowfield, Velikova and Sharpe, 2008). The differences in expected test scores between the four mode groups were calculated following Chalmers, Counsell and Flora (2016). For this, differences in the test score functions were calculated between any two of the four mode groups while one is arbitrarily chosen as the reference group. The differences represent the mode-specific biases in total scores and are given in the raw score metric (i.e., number correct scores). They are referred to as the signed DTF statistics $\hat{sDTF}$. In the present study, *sDTF* can range from – 36 to 36 (i.e., the highest possible test scores). Negative values indicate that the values of the reference group scores are on average lower than those of the comparison group, despite holding the proficiency distributions in both groups constant. In contrast, positive values indicate higher scores in the reference group. The unsigned DTF statistic $\hat{uDTF}$ represents the absolute difference between test response curves and, thus, can range from 0 and 36. It quantifies the size of the difference but not its direction. Next to the bias in the raw score metric, we also give the percentage bias ${\hat{uDTF}}_{\%}$ as the relative increase in test scores for the comparison group (as compared to a reference group). Finally, $\hat{sDTF}$, $\hat{uDTF}$, and ${\hat{uDTF}}_{\%}$ were evaluated for the whole sample and also across specific regions of the latent variable to examine whether mode effects are more pronounced, for example, among low proficient respondents. In contrast to traditional DTF analyses that treat item parameters as known values and, typically, ignore that they are sample estimates (e.g., Raju et al., 1995), we acknowledged parameter uncertainty in our analyses by repeating the DTF analyses 100 times for different item parameters that were randomly drawn based on the asymptotic variance-covariance matrix of the parameter estimates (see Chalmers et al., 2016). In this way, we were able to account for parameter uncertainty and construct confidence intervals for the DTF statistics to quantify their precision.

#### Analyses of prediction bias

To analyze prediction bias, we estimated linear regressions with either grade point average, academic self-concept, helplessness, or intention to quit as a criterion. The focal scientific literacy scores, the assessment mode (dummy-coded with PBA as reference category), and the respective interactions were used as predictors. Moreover, gender (coded – 0.5 = male, 0.5 = female) and study type (coded – 0.5 = teacher education, 0.5 = other subjects) were included as control variables because these variables showed mode-specific selection effects (see below). Significant interaction effects indicate a mode-specific prediction bias. To make parameter estimates comparable, scientific literacy scores and the criterion variables were *z*-standardized. Missing values in the criteria were imputed 20 times by chained equations (van Buuren & Groothuis-Oudshoorn, 2010). As mentioned before, the analyses of prediction bias (*RQ3*) were limited to a subsample of *n* = 1,825 students that were enrolled in natural science courses.

### Open practices

This paper uses data from the NEPS (see Blossfeld et al., 2011). The anonymized data including information on the administered tests are available at 10.5157/NEPS:SC5:12.0.0. The data collection procedure is described in Prussog-Wagner et al. (2013). Moreover, the analysis syntax to reproduce our results can be found at https://github.com/bieneSchwarze/ModeEffectsInNEPS.

## Results

### Mode-specific self-selection bias

The mode-specific response rates are summarized in Table 1. Contrary to previous research, students who were randomly assigned to the modes showed notably higher response rates in unstandardized and unsupervised web-based assessments (54.2%) as compared to standardized and supervised assessments: PBA (25.6%) and CBA (18.2%). Moreover, PBA and CBA non-responders that were switched to WBA showed a response rate of 25.6%. Thus, the flexibility in location and time offered by web-based assessments seems to be of particular importance to university students, resulting in a substantial participation rate in the WBA mode after having refused PBA or CBA testing.

**Table 1:** Number of students by mode and test participation

Mode	Group	Number of students with a test assigned	Number of students conducting the test	Response rate
PBA	Random assignment	5371	1374	25.6%
CBA	Random assignment	3431	623	18.2%
WBA	Random assignment	8671	4701	54.2%
	Non-responders from PBA or CBA	6804^†^	1744	25.6%

*Note*. PBA = standardized and supervised paper-based assessment; CBA = standardized and supervised computer-based assessment; WBA = unstandardized and unsupervised web-based assessment. ^†^ One case is missing because the person was not a non-responder in PBA or CBA mode, but the person gave too few valid responses for the estimation of a valid competence score (see Pohl & Carstensen, 2013)

**Fig. 3:**
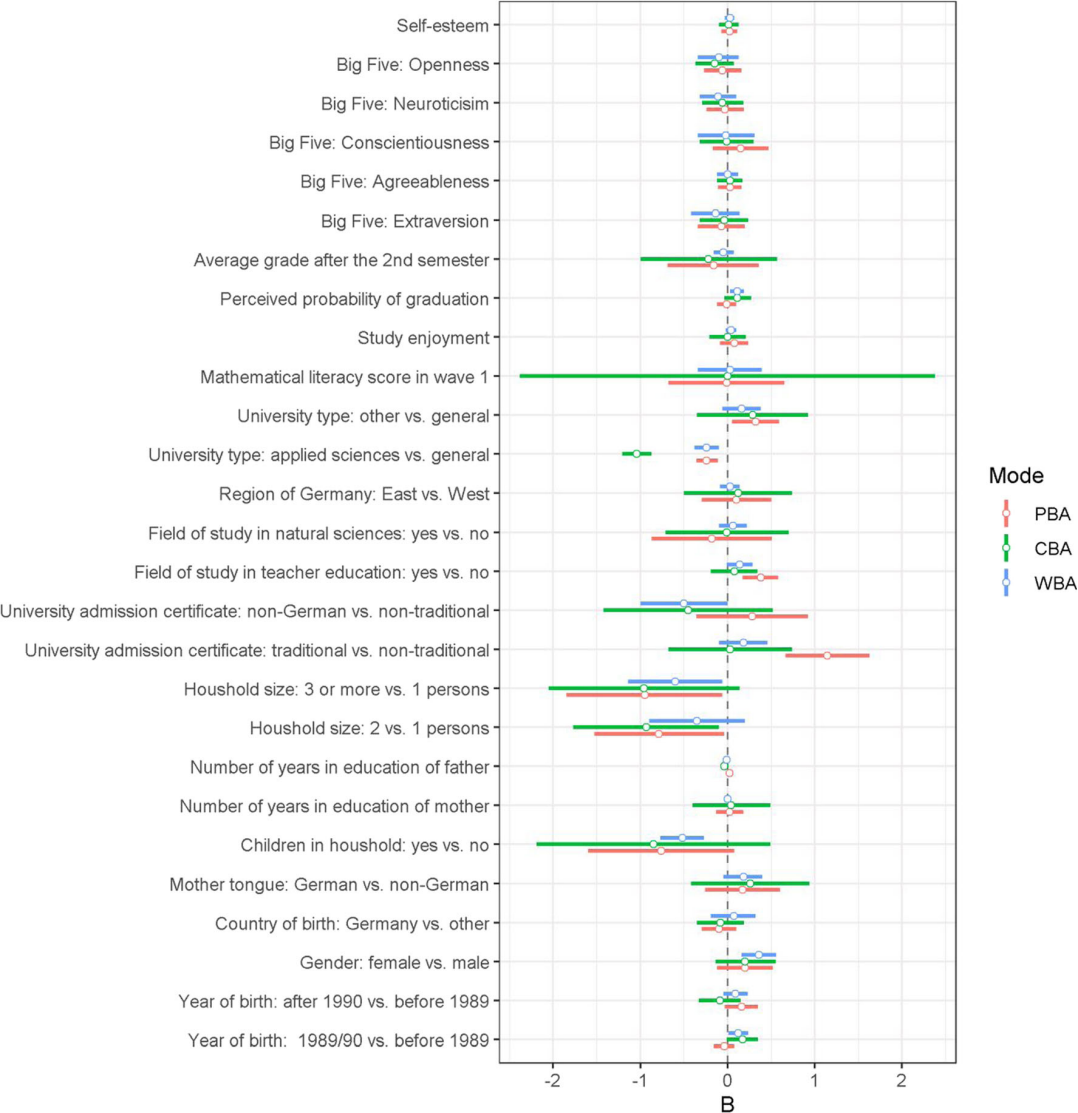
Estimated mode-specific main effects *B* (*white dots*) of self-selection analysis for paper-based assessments (PBA), computer-based assessments (CBA), and web-based assessments with random assignment (WBA) with 95% confidence intervals (*horizontal bars*). *Note*. Dependent variable is response (coded as 1 = response and 0 = nonresponse). An effect is significant at the .05 level if the confidence interval does not cross the vertical line

The results of our analyses whether unsupervised WBA introduced a different selection bias as compared to supervised PBA or CBA regarding several socio-demographic, personality, and student characteristics are given in Fig. 3. The figure depicts the conditional (i.e., main) effects from the related logit model (significant effects have 95% confidence intervals that do not cross the vertical zero line). For example, we found that female students had a significantly (*p* < .05) stronger tendency than male students to participate in WBA (*B* = 0.36, 95% CI [0.17, 0.55]), whereas the respective effect was slightly smaller (and non-significant) in PBA (*B* = 0.19, 95% CI [– 0.16, 0.54]) or CBA (*B* = 0.18, 95% CI [– 0.17, 0.53]). However, the difference in effects between modes (i.e., the interaction effect) was not significantly different from zero, *B* = – 0.17, 95% CI [– 0.54, 0.20] for PBA versus WBA and *B* = – 0.17, 95% CI [– 0.53, 0.18] for CBA versus WBA (see supplement material for detailed results). Thus, the selection effect resulting from students’ gender was similar in all three assessment modes. Similarly, students with children generally showed a significantly lower tendency to participate in the study in WBA (*B* = – 0.50, 95% CI [– 0.78, – 0.22]), whereas the respective effects were not significant for PBA (*B* = – 0.79, 95% CI [– 1.65, 0.06]) and CBA (*B* =– 0.88, 95% CI [– 2.27, 0.51]). Again, the differences in effects between modes were not significant (*B* = – 0.29, 95% CI [– 0.94, 0.36] for PBA versus WBA and *B* = – 0.38, 95% CI [– 1.36, 0.61] for CBA versus WBA). We found only two significant (*p* < .05) mode-specific effects on students’ participation (see supplement material). First, students at universities of applied sciences had a significantly lower tendency to participate in the supervised CBA as compared to the unsupervised WBA mode (*B* = – 0.79, 95% CI [– 1.19, – 0.39]). In absolute terms, the respective (main) effect was substantially larger for CBA, *B* = – 1.02, 95% CI [– 1.25, – 0.80], as compared to WBA, *B* = – 0.23, 95% CI [– 0.37, – 0.10]. Second, students with a non-traditional German university admission certificate (e.g., a completed vocational training) showed a significantly higher tendency towards participating in PBA than in WBA as compared to students with a traditional German university admission certificate with *B* = 1.00, 95% CI [0.24, 1.72]). The (related main) effect was significant for PBA, *B* = 1.18, 95% CI [0.68, 1.68], but not for WBA, *B* = 0.18, 95% CI [– 0.09, 0.45]. The remaining variables showed no significant mode-specific selection effects.

In summary, these analyses showed that the willingness of students to participate in unsupervised web-based cognitive testing depended on different background characteristics. For the attendance of universities of applied sciences and general universities and the university admission certificate, the respective effects were systematically related to specific assessment modes namely to CBA and PBA, not so much to WBA (*RQ1*). However, it needs to be stressed that the observed mode-specific selection bias was small. It contributed only an addition of about 1% of variance explained to the (selection) model comprising solely main effects (see supplement material). We conducted nonresponse adjustments for the following analyses to acknowledge mode-specific selection effects and we derived respective propensity scores. The respective results are described in the supplement material.

### Mode-specific measurement bias

The one-parametric item response models fitted to the responses in each of the four mode groups exhibited satisfactory item fits, with all weighted mean square statistics falling below the recommended threshold of 1.20. The population variances in scientific literacy were slightly larger in PBA (*Var* = 0.73) as compared to the three computerized modes (*Var*s between 0.57 and 0.64; see Table 2), thus, reflecting interindividual differences in scientific literacy more strongly. The respective empirical reliability estimates (Adams, 2005) showed similar measurement precisions in both supervised settings (*Rel* = .79 /.80), whereas the two WBA conditions exhibited lower reliabilities (*Rel* = .71 /.70). Thus, unstandardized and unsupervised testing resulted in larger measurement errors as compared to standardized and supervised assessments.

**Table 2:** Latent variances and empirical reliabilities by assessment mode

	PBA	CBA	WBA	WBA-switch
Variance	0.73 [0.69, 0.78]	0.59 [0.52, 0.66]	0.63 [0.59, 0.67]	0.57 [0.53, 0.60]
Reliability	.79	.80	.71	.70

*Note*. Latent population variances (with 95% confidence intervals) and empirical WLE reliabilities (see Adams, 2005). PBA = standardized and supervised paper-based assessment, CBA = standardized and supervised computer-based assessment, WBA = unstandardized and unsupervised web-based assessment with random assignment, WBA-switch = unstandardized and unsupervised web-based assessment with non-random assignment (for PBA / CBA non-responders)

**Fig. 4:**
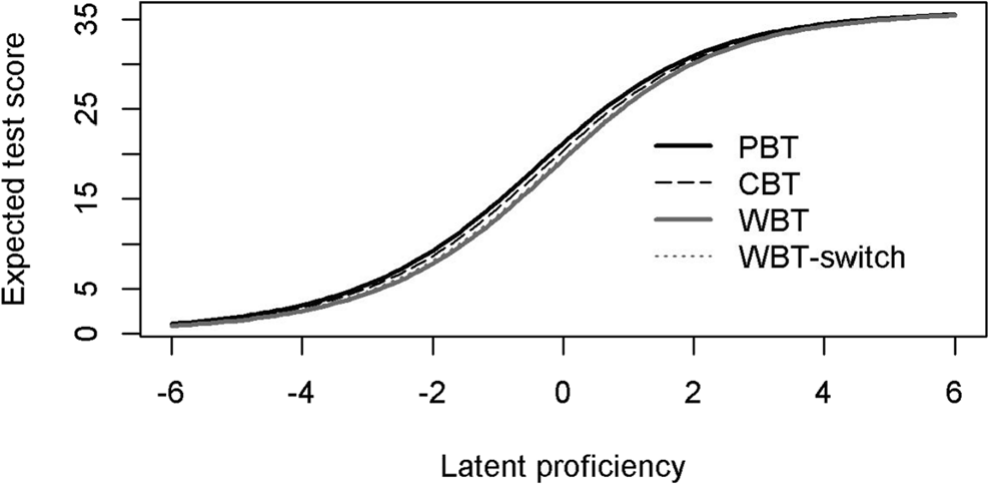
Test scoring functions for paper-based assessments (PBA), computer-based assessments (CBA), web-based assessments with random assignment (WBA), and web-based assessment with non-random assignment (WBA-switch)

**Fig. 5:**
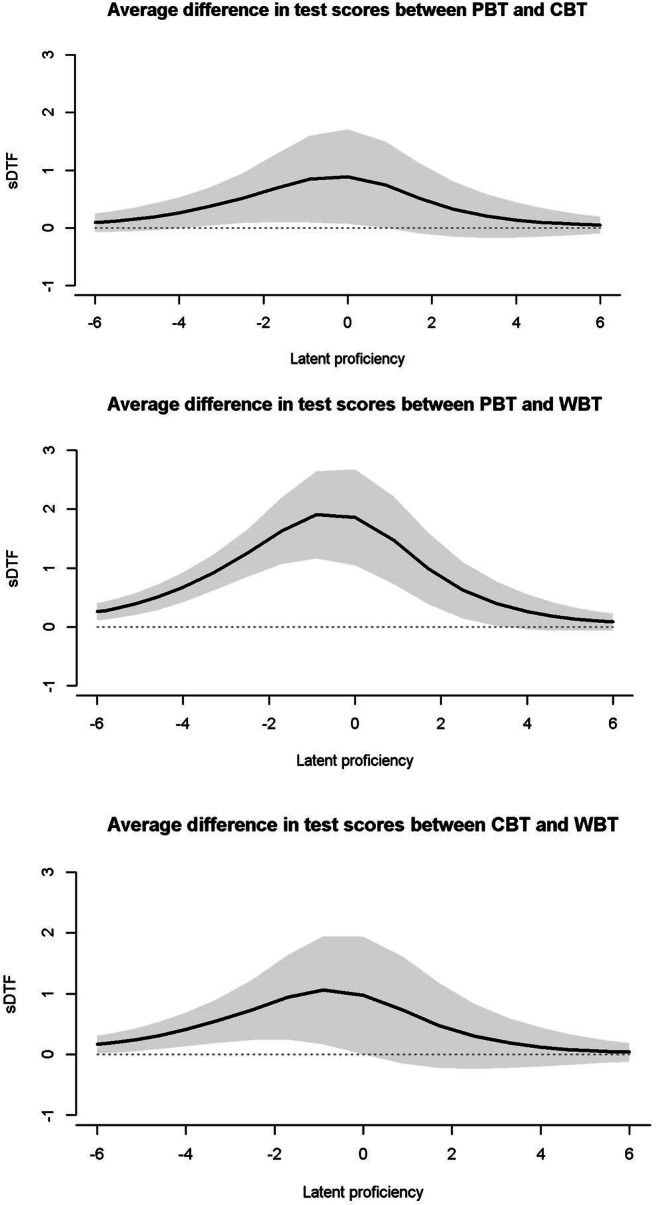
Signed differential test functioning (sDTF) for paper-based assessments (PBA), computer-based assessments (CBA), and web-based assessments (WBA) with 95% confidence intervals

Whether the assessment mode introduced a systematic bias into the measured constructs was evaluated using DTF analyses. The model-implied test scoring functions for the four mode groups are presented in Fig. 4. These highlight only rather small differences between groups. Given the same proficiency, respondents tended to achieve more points in supervised (particularly, paper-based) settings as compared to unsupervised WBA. The two WBA comparisons also showed no notable differences. The $\hat{sDTF}$ quantified these differences as 0.87 for PBA (reference group) versus WBA and as 0.48 for CBA (reference group) versus WBA (see Table 3). Thus, on average, WBA resulted in a rather small (albeit significant) bias leading to a difference of less than one point in test scores as compared to PBA or CBA. Interestingly, mode effects (i.e., paper versus computer) and setting effects (i.e., supervised and standardized versus unsupervised and unstandardized) each contributed a similar share of bias in WBA (about 0.40 to 0.50 raw score points). Respondents that were redirected to WBA after having refused to participate in supervised assessments (WBA-switch) did not generate a significant (*p* < .05) bias as compared to the random WBA sample. Analyses examining the absolute differences in test scores ($\hat{uDTF}$) resulted in highly similar results, indicating that the bias in test scores consistently fell in the same direction: Overall, the bias introduced by WBA was small and amounted to about 1.0–2.5% of the total score (see Table 3). Finally, we also evaluated whether measurement bias might be more pronounced at different levels of the latent proficiency. Figure 5 shows that $\hat{sDTF}$ was larger at lower to medium levels of scientific literacy and grew smaller for very high proficiencies. However, the respective confidence intervals showed that $\hat{sDTF}$ between PBA and CBA was only significant (*p* < .05) at about one to three standard deviations below the mean. In total, the largest bias between PBA (reference group) and CBA amounted to 0.85 raw score points only. In contrast, the respective bias was more pronounced between PBA (reference group) and WBA. $\hat{sDTF}$ was significant along most of the proficiency scale, except for very high competences about three standard deviations above the mean. The largest bias was observed at one to two standard deviations below the mean and peaked at about 1.91 raw score points. Although these differences are relatively small, they highlight that respondents were differently affected by the assessment mode depending on their latent proficiency (*RQ2*).

**Table 3:** Average differential test functioning

	$$ \hat{sDTF} $$					
	CBA	WBA	WBA-switch			
PBA	0.39 [0.05, 0.72]	0.87 [0.56, 1.17]	0.72 [0.39, 1.05]			
CBA		0.48 [0.16, 0.80]	0.34 [– 0.00, 0.67]			
WBA			– 0.14 [– 0.44, 0.16]			
WBA-switch						
	$\hat{uDTF}$			$\hat{uDT{F}_{\%}}$		
	CBA	WBA	WBA-switch	CBA	WBA	WBA-switch
PBA	0.40 [0.10, 0.71]	0.87 [0.57, 1.17]	0.73 [0.41, 1.05]	1.12% [0.27%, 1.97%]	2.42% [1.58%, 3.25%]	2.02% [1.13%, 2.91%]
CBA		0.50 [0.21, 0.78]	0.37 [0.09, 0.64]		1.39% [0.59%, 2.18%]	1.02% [0.25%, 1.79%]
WBA			0.22 [0.02, 0.42]			0.61% [0.06%, 1.16%]
WBA-switch						

*Note*. Confidence intervals (95%) are given in parentheses. $\hat{sDTF}$ = average difference in test scores between groups, $\hat{uDTF}$ = average absolute difference in test scores between groups, $\hat{uDT{F}_{\%}}$ = $\hat{uDTF}$ as percentage of maximum test score (here: 36 points), PBA = standardized and supervised paper-based assessment, CBA = standardized and supervised computer-based assessment, WBA = unstandardized and unsupervised web-based assessment with random assignment, WBA-switch = unstandardized and unsupervised web-based assessment with non-random assignment (for PBA / CBA non-responders). Rows represent the reference groups with positive values indicating higher scores in these groups

### Mode-specific prediction bias

Whether the assessment mode affected the associations between scientific literacy and different variables measured about 6 months later was examined using linear regression analyses (see Table 4). For three of the four examined criteria, the expected associations with scientific literacy were observed with standardized regression weights of *B* = 0.21, 95% CI [0.08, 0.34], for grade point average, *B* = 0.46, 95% CI [0.34, 0.59], for academic self-concept, and *B* = – 0.22, 95% CI [– 0.34, – 0.09], for study-related helplessness. In contrast, for intention to quit no significant (*p* < .05) effect was found, *B* = – 0.09, 95% CI [– 0.25, 0.06]. More importantly, we found no significant moderating effects of the assessment mode for three of these outcomes. Thus, the predictions of grade point average, helplessness, and intention to quit were not affected by how scientific literacy was measured (see Table 4). In contrast, for academic self-concept significant (*p* < .05) moderating effects were found. The association between self-concept and scientific literacy was smaller in CBA (*B* = 0.13), WBA (*B* = 0.10), and WBA-switch (*B* = 0.20) as compared to PBA (*B* = 0.46). This suggests that prediction bias might be construct-specific and a lack of prediction bias for a specific outcome cannot be generalized to different outcomes (*RQ3*). It should be noted that the prediction models as a whole explained only a very small proportion of variance in the outcome variables, namely only between 3 to 5%. Furthermore, although we observed significant moderation effects of the PBA mode concerning academic self-concept, the additional consideration of this moderation effect leads to less than 1% additional variance explained.

**Table 4:** Linear regressions evaluating prediction bias

	Criterion:	Grade point average		Academic self-concept		Study-related helplessness		Intention to quit	
	Predictor	*B*	95% CI	*B*	95% CI	*B*	95% CI	*B*	95% CI
	Intercept	0.15*	(0.00, 0.30)	– 0.19*	(– 0.33, – 0.06)	0.14*	(0.01, 0.28)	– 0.01	(– 0.17, 0.14)
*Main effect of science*									
1.	Scientific literacy	0.21*	(0.08, 0.34)	0.46*	(0.34, 0.59)	– 0.22*	(– 0.34, – 0.09)	– 0.09	(– 0.25, 0.06)
*Main effects of mode*									
2.	CBA	– 0.21	(– 0.44, 0.03)	0.29*	(0.08, 0.51)	– 0.06	(– 0.27, 0.16)	0.07	(– 0.15, 0.28)
3.	WBA	– 0.07	(– 0.25, 0.11)	0.17	(0.00, 0.34)	– 0.11	(– 0.28, 0.05)	0.05	(– 0.14, 0.24)
4.	WBA-switch	– 0.11	(– 0.32, 0.09)	0.09	(– 0.10, 0.28)	– 0.09	(– 0.28, 0.10)	0.18	(– 0.04, 0.39)
*Moderating effects*									
5.	1. x 2.	– 0.09	(– 0.30, 0.11)	– 0.33*	(– 0.56, – 0.11)	0.01	(– 0.19, 0.20)	0.09	(– 0.17, 0.35)
6.	1. x 3.	– 0.13	(– 0.29, 0.03)	– 0.36*	(– 0.52, – 0.20)	0.09	(– 0.06, 0.23)	– 0.05	(– 0.23, 0.14)
7.	1. x 4.	– 0.04	(– 0.23, 0.15)	– 0.26*	(– 0.45, – 0.08)	0.09	(– 0.09, 0.28)	– 0.12	(– 0.33, 0.09)
	*Covariates*								
8.	Sex	– 0.22*	(– 0.35, – 0.10)	0.10	(– 0.02, 0.23)	– 0.05	(– 0.19, 0.09)	– 0.15*	(– 0.28, – 0.01)
9.	Teacher education	– 0.09	(– 0.23, 0.05)	– 0.05	(– 0.18, 0.08)	– 0.01	(– 0.15, 0.13)	0.01	(– 0.12, 0.14)
	*R*^2^ / Δ*R*^2^	.03 / .01		.05 / .01		.03 / .00		.03 / .00	

*Note*. Limited to students of natural sciences (*N* = 1,825). Linear regression of outcomes on science literacy, assessment mode (dummy-coded with PBA as reference), respective interactions, and covariates. Sex (– 0.5 = male, 0.5 = female) and teacher education (0.5 = other study, – 0.5 = teacher education) were effect-coded. Outcomes and scientific literacy were *z*-standardized. *R*^2^ / Δ*R*^2^ = Explained variance / incremental variance explained by moderating effects. PBA = standardized and supervised paper-based assessment, CBA = standardized and supervised computer-based assessment, WBA = unstandardized and unsupervised web-based assessment with random assignment, WBA-switch = unstandardized and unsupervised web-based assessment with non-random assignment (for PBA / CBA nonresponders)

*p < .05

## Discussion

The way cognitive abilities are measured in large-scale studies can influence their validity and, thus also the conclusions drawn on substantial research questions that are investigated by these measures (e.g., Lenhard et al., 2017; Robitzsch et al., 2017). This study examined to what degree cognitive assessments in unsupervised and unstandardized web-based settings are comparable to supervised and standardized settings. In contrast to previous research (e.g., Al Baghal, 2019; Schroeders & Wilhelm, 2011), we adopted a multi-perspective approach and evaluated selection bias, measurement bias, and prediction bias of WBA among a large sample of third-year university students. These analyses yielded three main results:

First, the response rate of 54% in unsupervised WBA was about 2–3 times higher as compared to supervised CBA or PBA. Moreover, for non-responders in the supervised settings, a response rate of 26% was observed for a subsequent WBA switch option. Thus, mixed-mode designs including a web-based component seem to be an effective way to increase response rates. These findings are in contrast to related meta-analytic summaries that found substantially lower response rates in web-based (self-report) surveys for general populations (for a respective meta-analysis see Daikeler et al., 2020). Although this meta-analysis also noted that students seemed to be less affected by the survey mode and highlighted only marginal differences between supervised and unsupervised settings, an advantage of WBA in terms of response rates has not yet been systematically observed. Our study suggests that web-based testing is particularly attractive for time-consuming and cognitively demanding tasks such as the scientific literacy test administered in the considered case. Highly mobile and difficult to reach respondents with limited time resources such as university students might find the liberty of choosing when and where to take a test appealing and, thus, are more inclined to participate in unsupervised assessments. More importantly, we found only little evidence for pronounced selection effects in WBA and other modes. Only the kind of university (university of applied sciences versus general university) and the kind of university admission certificate (traditional versus non-traditional) had an impact on students’ mode-specific propensities, but more in relation to PBA and CBA than to WBA. Therefore, it is unlikely that WBA or mixed-mode designs including a web-based component result in substantially biased samples as compared to PBA or CBA.

Second, in line with previous research (e.g., Mead & Drasgow, 1993; Wang et al., 2007, 2008), we found that the measurement properties of the administered instrument were not substantially affected by the assessment modes, even after correcting for selection effects. The most pronounced effect was observed for the reliability estimates. These were lower in WBA compared to PBA and CBA. This finding might be a consequence of environmental distractions in unsupervised WBA if participants’ attention is redirected during the test by, for example, phone calls, instant messages, conversations, or loud music (cf. Hardré, Crowson and Xie, 2012; Zwarun and Hall, 2014). The lower reliability estimates might also be caused by motivational differences: Participants in the web-based mode may not invest the same effort to respond to cognitively demanding items as when tested in PBA or CBA mode (see Finn, 2015; Wise, 2006). For example, it has been shown that later testing times (e.g., in the afternoon) were associated with lower effortful responding (Wise, Ma, Kingsbury and Hauser, 2010). Since respondents in WBA are free to choose the time of assessment, it is conceivable that differences in mental fatigue may have contributed to the observed mode difference. Moreover, the presented analyses highlighted a systematic measurement bias resulting in lower test scores in CBA and WBA as compared to PBA. Similar findings have been previously reported: the computerization of competence tests in large-scale studies seems to (slightly) increase the test difficulty (see Robitzsch et al., 2017). However, the respective bias did not seem to be substantial and fell at less than one score point (i.e., about 1–2.5% of the total score). More worrying is the fact that the respective bias was not constant across the latent proficiency scale. It was more pronounced at low to medium ability levels. Thus, in mixed-mode designs, unsupervised web-based testing systematically disadvantages low and medium performing respondents and contributes to unfair measurements. As long as population effects are the focus of interest such as in educational large-scale assessments, these distortions might be negligible. However, they might be more serious if the competences of individuals are compared.

Third, scientific literacy scores were associated with different outcomes measured 6 months after the cognitive assessment. Importantly, the mode of administration did not impact on the prediction of grade point average, study-related helplessness, and intention to quit the study program. In other words, whether scientific literacy was measured on paper or computer and in standardized or in unstandardized settings was immaterial for its predictive validity. These results are in line with corresponding findings from non-cognitive employment testing that found comparable predictive validities in paper-based and web-based surveys (Beaty et al., 2011). However, we observed significantly different effects for academic self-concept which yielded higher associations in PBA as compared to the computerized testing modes. The reason for this discrepancy is unclear. In summary, our results highlight that comparable predictive validities across different assessment modes should not be taken for granted. On the contrary, there is a clear indication that differential effects need to be scrutinized separately for each criterion examined before cognitive scores from different modes can be combined and analyzed.

### Implications and recommendations

The presented findings from a mode experiment among German university students suggest that web-based cognitive assessments are a feasible option in large-scale studies. Particularly, if university students are the target population WBA might counteract the problem of decreasing response rates (see Beullens, Vandenplas, Loosveldt and Stoop, 2018). Moreover, we strongly recommend offering university students who did not respond to CBA or PBA the option of switching to WBA, as this significantly increases response rates without adversely affecting the measurement quality. Nonetheless, in mixed-mode designs, the switch from supervised paper-based testing to unsupervised computerized testing is accompanied by a systematic bias resulting in lower scores in WBA. Although the respective bias seems to be small, it is advisable to implement explicit link studies (cf. Fischer, Gnambs, Rohm and Carstensen, 2019) that allow correcting for the observed mean level difference and placing cognitive scores from different modes on a common scale. Overall, the reported findings suggest weak mode-specific effects which should encourage researchers to seriously consider the less costly self-administered web-based modes in cognitive large-scale assessments, at least for studies in higher education.

### Limitations and directions for future research

The presented results offer several opportunities for refinement and extension. First, our findings pertain to a specific test and target population. Whether these results can be generalized to other cognitive instruments (e.g., fluid measures of intelligence), to other mobile devices (e.g., smartphones, tablets) and, particularly, non-student samples need to be explored in similar high-powered mode experiments. In particular, it is conceivable that the observed advantage of WBA concerning response rates is specific for technology-literate respondents such as university students and WBA is not as effective in samples from the general population (cf. Daikeler et al., 2020). Moreover, our research is silent on differences in the response processes between modes. For example, respondents might adopt rather different response strategies resulting in, for example, mode-specific response latencies or test interruptions. Analyses of non-reactive process data might give further insights into how people handle cognitive tests in different administration modes (cf. Hahnel et al., 2019). Finally, the study represents a snapshot at one point in time. With the increasing use of digital media in respondents’ work and private lives, web-based cognitive tests are likely to become more common. It is, therefore, important to monitor whether the observed mode effects change over time and whether there may even be additional benefits of WBA in the near future.

### Constraints of generality

The results of the presented mode experiment highlighted few notable differences between supervised paper-based or computerized and unsupervised web-based cognitive assessments. However, the generalizability of these findings might be constrained by three major aspects: First, our study used a sample of university students that usually exhibit unique cognitive, socio-emotional, and behavioral patterns as compared to the general population (e.g., Fosgaard, 2020; Hanel and Vione, 2016). Because students in higher education, on average, exhibit higher cognitive skills, more pronounced differential test functioning among low-achievers might be expected in more diverse samples that cover the whole ability range. Thus, the optimistic conclusions regarding web-based large-scale assessments might not extend to representative samples of adolescents (e.g., PISA) or adults (e.g., PIAAC). Second, the reported results refer to a specific test of scientific literacy. Although science represents a core domain that is addressed in many international large-scale assessments, these findings should not be readily generalized to other domains. Mode effects might be test-specific and have more substantial consequences, for example, for the assessment of reading skills (Delgado et al., 2018). Third, the web-based condition implemented in the present study referred to tests administered on notebooks and personal computers. Assessments with smartphones or tablets were not considered. Given the different conditions under which these devices present the test material and the tests have to be performed (e.g., by touching on a screen instead of typing), the conclusions regarding the feasibility of web-based testing in large-scale studies cannot easily be generalized to these applications.

## Conclusions

Web-based cognitive assessments represent an intriguing opportunity to collect cost-efficient and timely data from a large sample of respondents. At least university students are more likely to participate in related studies without introducing a substantial bias. The different assessment modes are not strictly equivalent in terms of selection effects and measurement quality. Nonetheless, corresponding distortions seem to be small and thus lead to a negligible bias in large-scale cognitive studies.

## Electronic supplementary material

ESM 1 (PDF 292 kb)

## Footnotes

1 Educational large-scale studies such as PIAAC or the NEPS represent low-stakes assessments that are voluntary and do not yield personal consequences for the test takers. In contrast, medium- or high-stakes assessments such as school exams, university admission tests, or certification programs can have a great impact on the test takers life and, thus, can exhibit different response behaviors (e.g., Azmat, Calsamiglia and Iriberri, 2016; Jalava, Joensen and Pellas, 2015).

2 Alternatively, repeated measurement designs might be adopted that assign all participants to all modes one after the other. Then, the cognitive scores obtained under different modes can be compared. However, this approach is only meaningful for constructs for which memory effects are unlikely or when using complex booklet designs that prevent repeated item presentations to the same test takers (see Kroehne, Buerger, et al., 2019).

3 About half of the students in the paper-based condition used digital pens to note their answers on the response sheet to also record their response times, whereas the remaining sample used ordinary pens. Because this distinction was not relevant for the present research, both groups were combined in our analyses.

4 In WBA, the respondents were unable to access the web-based tests using smartphones or mobile devices with small screens. All students finished the competence test on their private notebooks or personal computers. Detailed information on the respondents’ computer system (e.g., computer type, screen size) that was used to access the web-based test was not collected because of privacy concerns.

5 Here, students who neither participated in the PBA nor the CBA and who were later invited to switch to the WBA mode were not considered. The reason is, that they are a selective, nonrandom group themselves.

6 Indicators on respondents’ participation behaviors in previous NEPS waves were not considered because these would not inform about the impact of individual characteristics and learning environment on mode-specific self-selection. However, to increase the prediction accuracy the respective information was included in the nonresponse analyses to derive the mode-specific participation probabilities (and thus the propensity scores compensating for nonrandom nonresponse).
