# Supplementary material for: Web-based and mixed-mode cognitive large-scale assessments in higher education: An evaluation of selection bias, measurement bias, and prediction bias
Source: Behav Res Methods. 2020 Oct 1;53(3):1202–17. doi: 10.3758/s13428-020-01480-7 (PMC8219565; doi:10.3758/s13428-020-01480-7)
Supplement: Supplementary file 1 — (PDF 292 kb) [file 13428_2020_1480_MOESM1_ESM.pdf]

Supplement Material for

Web-Based and Mixed-Mode Cognitive Large-Scale Assessments:

An Evaluation of Selection Bias, Measurement Bias, and Prediction Bias

- Predictors of nonresponse
- Table S1. Summary statistics of predictors of self-selection and nonresponse by mode.
- Table S2. Logit regression for self-selection by assessment mode
- Table S3. Logit regressions for nonresponse analyses for students participating in the mode-specific assessments.

Predictors of Nonresponse

We acknowledged 28 variables as potential predictors of nonresponse (see Table S1). As sociodemographic variables we considered students' year of birth (0 = "before 1989", 1 = "in 1989 or 1990", 2 = "after 1990"), gender (0 = "male", 1 = "female"), country of birth (0 = "not Germany", 1 = "Germany"), mother tongue (0 = "non-German", 1 = "German"), whether children lived in the household (0 = "no", 1 = "yes"), the number of years in education of their mothers and fathers, and their household size at the time of the testing (0 = "one person", 1 = "two persons", 2 = "three or more persons"). These or similar variables have been previously found to explain wave-specific participation propensities in the NEPS (Zinn, Steinhauer, & Aßmann 2017). Therefore, we considered them suitable candidates for mode-specific nonresponse predictors. The same applies to the following study-specific variables: does a student has a traditional university admission certificate[1] (0 = "no, a non-traditional German one", 1 = "yes, a traditional German one", 2 = "yes, a non-German one"), is the student's study field teacher education (0 = "no", 1 = "yes"), or natural sciences (0 = "no", 1 = "yes"), where does he /she study (0 = "Western Germany", 1 = "Eastern Germany"), is the student's university a public or private one (0 = "private", 1 = "public"), and is the university one of applied sciences or a general one or of some other kind (1 = "general university", 1 = "university of applied sciences", 2 = "of some other kind"). Besides, we took into account factors for academic success. We considered students' competence scores in mathematics obtained in the first wave of the NEPS (see Gerken & Schnittjer, 2017), their subjective probability of successful graduation in that year (on a scale from 1 to 5 with 1 = "very unlikely" and 5 = "very likely"), and whether they enjoyed studying (on a scale from 1 to 5 with 1 = "does not apply at all" and 5 = "is absolutely true"). Furthermore, our nonresponse model contained students' average grades in their study programs after their first year, i.e.

[1] The German notions for the traditional university admission certificates are *Abitur* or *Fachabitur*.

after their 2nd semester. In addition, we included in our nonresponse models measures of the students' Big Five personality traits, that is, extraversion, agreeableness, conscientiousness, neuroticism, and openness (Rammstedt & John, 2007) and their self-esteem (Rosenberg, 1965). To map the general willingness of the students to participate in surveys we used information concerning their participation in previous NEPS waves. We used as predictors dummy variables indicating their participation in the paper-based competence tests at wave 1 in 2011 (0 = "no", 1 = "yes"), in the web-based interviews at the waves 2 and 4 in 2011 and 2012 (0 = "no", 1 = "yes"), and in the telephone interview at wave 3 in 2012 (0 = "no", 1 = "yes").

References

Gerken, A.-L., & Schnittjer, I. (2017): *NEPS Technical Report for Mathematics: Scaling Results of Starting Cohort 5 for First-Year Students* (NEPS Survey Paper No. 17). Bamberg, Germany: Leibniz Institute for Educational Trajectories, National Educational Panel Study.

Rammstedt, B., & John, O. P. (2007). Measuring personality in one minute or less: a 10-item short version of the Big Five Inventory in English and German. *Journal of Research in Personality*, *41*, 203-212. https://doi.org/10.1016/j.jrp.2006.02.001

Rosenberg, M. (1965). *Society and the Adolescent Self-Image*. Princeton, NJ: Princeton University Press.

Zinn, S., Steinhauer, H. W., & Aßmann, C. (2017). *Samples, weights, and nonresponse: the student sample of the National Educational Panel Study (Wave 1 to 8)* (NEPS Survey Paper No. 18). Bamberg, Germany: Leibniz Institute for Educational Trajectories, National Educational Panel Study.

Table S1.

Summary Statistics of Predictors of Self-Selection and Nonresponse by Mode.

	Variable	Reference category	PBA			CBA			WBA			WBA-switch		
			M	*SD*	MI	*M*	*SD*	MI	*M*	*SD*	MI	*M*	*SD*	MI
Socio-demographics	Gender	male	0.42	--	0.00	0.33	--	0.00	0.41	--	0.00	0.40	--	0.00
	female		0.58	--	0.00	0.67	--	0.00	0.59	--	0.00	0.60	--	0.00
	Country of birth	not Germ.	0.06	--	0.00	0.05	--	0.00	0.07	--	0.00	0.06	--	0.00
	Germany		0.94	--	0.00	0.95	--	0.00	0.93	--	0.00	0.94	--	0.00
	Mother tongue	non-Germ.	0.08	--	0.00	0.06	--	0.00	0.09	--	0.01	0.08	--	0.01
	German		0.92	--	0.00	0.94	--	0.00	0.91	--	0.01	0.92	--	0.01
	Children	no	0.97	--	48.09	0.98	--	46.16	0.94	--	47.01	0.96	--	49.10
	yes		0.03	--	48.09	0.02	--	46.16	0.06	--	47.01	0.04	--	49.10
	Household size	one person	0.46	--	26.75	0.44	--	24.25	0.48	--	29.11	0.48	--	31.36
	two persons		0.20	--	26.75	0.19	--	24.25	0.20	--	29.11	0.18	--	31.36
	three + persons		0.34	--	26.75	0.37	--	24.25	0.32	--	29.11	0.34	--	31.36
	Birth year	before 1989	0.23	--	0.00	0.21	--	0.00	0.28	--	0.00	0.23	--	0.00
	1989/1990		0.52	--	0.00	0.53	--	0.00	0.48	--	0.00	0.52	--	0.00
	after 1990		0.25	--	0.00	0.26	--	0.00	0.24	--	0.00	0.25	--	0.00
	Years in education of mother		14.12	2.37	2.42	14.23	2.43	1.60	14.13	2.42	2.68	14.11	2.40	2.41
	Years in education of father		14.61	2.37	3.78	14.77	2.55	2.54	14.50	2.55	3.74	14.63	2.54	3.73
Personality	Extraversion		3,72	0.83	26.33	3.77	0.81	23.78	3.71	0.85	25.42	3.74	0.82	29.42
	Agreeableness		3.57	0.55	26.29	3.66	0.55	23.72	3.58	0.56	25.39	3.60	0.55	29.38
	Conscientiousness		3.77	0.76	26.25	3.83	0.75	23.72	3.78	0.76	25.38	3.77	0.76	29.37
	Neuroticism		2.71	0.82	26.27	2.71	0.80	23.72	2.70	0.80	25.37	2.70	0.82	29.38
	Openness		3.59	0.90	26.25	3.66	0.90	23.72	3.60	0.91	25.38	3.62	0.90	26.37
	Self-esteem		42.06	4.65	26.64	42.37	4.59	24.22	42.17	4.58	25.91	42.12	4.65	29.85
Student characteristics	Region of Germany	West.	0.76	--	0.04	0.95	--	0.03	0.74	--	0.01	0.84	--	0.04
	East		0.24	--	0.04	0.05	--	0.00	0.26	--	0.01	0.16	--	0.04
	Study of natural sciences	no	0.78	--	0.34	0.77	--	0.38	0.78	--	0.33	0.78	--	0.35
	yes		0.22	--	0.34	0.23	--	0.38	0.22	--	0.33	0.22	--	0.35
	Study of teacher education	no	0.71	--	0.24	0.56	--	0.23	0.69	--	0.24	0.68	--	0.25
	Yes		0.29	--	0.24	0.44	--	0.23	0.31	--	0.24	0.32	--	0.25

	Variable	Reference category	PBA			CBA			WBA			WBA-switch		
	Public university	no	0.02	--	0.00	0.00	--	0.00	0.04	--	0.01	0.01	--	0.00
	Yes		0.98	--	0.00	1.00	--	0.00	0.96	--	0.01	0.99	--	0.00
	Institution type	general	0.73	--	0.00	0.76	--	0.00	0.71	--	0.10	0.71	--	0.00
	Applied sciences		0.24	--	0.00	0.24	--	0.00	0.23	--	0.10	0.27	--	0.00
	other		0.03	--	0.00	0.00	--	0.00	0.06	--	0.10	0.02	--	0.00
	University admission certificate	non-traditional	0.29	--	26.72	0.25	--	24.13	0.32	--	29.04	0.33	--	31.29
	traditional		0.70	--	26.72	0.74	--	24.13	0.67	--	29.04	0.66	--	31.29
	non-German		0.01	--	26.72	0.01	--	24.13	0.01	--	29.04	0.01	--	31.29
	Average grade in 2nd semester		2.30	0.59	49.25	2.21	0.59	49.61	2.26	0.62	53.21	2.29	0.59	53.16
	Mathematical competence		-0.05	1.23	63.97	0.13	1.21	62.43	-0.04	1.22	69.25	-0.04	1.20	71.46
	Enjoy studying		4.32	0.79	0.15	4.30	--	0.26	4.27	0.83	0.46	4.29	0.82	0.22
	Probability of graduation		4.36	0.66	0.20	4.44	--	0.29	4.36	0.67	0.48	4.38	0.65	0.28
Participation in previous waves	PBA W1[(a)]	no	0.64	--	0.00	0.72	--	0.00	0.69	--	0.00	0.71	--	0.00
	yes		0.36	--	0.00	0.38	--	0.00	0.31	--	0.00	0.29	--	0.00
	CAWI W2[(b)]	no	0.31	--	0.00	0.39	--	0.00	0.31	--	0.00	0.35	--	0.00
	yes		0.69	--	0.00	0.71	--	0.00	0.69	--	0.00	0.65	--	0.00
	CATI W3[(c)]	no	0.26	--	0.00	0.34	--	0.00	0.25	--	0.00	0.29	--	0.00
	yes		0.74	--	0.00	0.76	--	0.00	0.75	--	0.00	0.71	--	0.00
	CAWI W4[(d)]	no	0.37	--	0.00	0.34	--	0.00	0.37	--	0.00	0.41	--	0.00
	yes		0.63	--	0.00	0.66	--	0.00	0.63	--	0.00	0.59	--	0.00
Sample size			5,371			3,431			8,671			6,804		

Note. PBA = standardized and supervised paper-based assessment, CBA = standardized and supervised computer-based assessment, WBA = unstandardized and unsupervised web-based assessment with random assignment, WBA-switch = unstandardized and unsupervised web-based assessment with non-random assignment (for PBA / CBA nonresponders), *M* = mean for continuous variables and the proportion for categorical variables, *SD* = standard deviation for continuous variables, MI = percentage of missing values. (a) PBA W1: paper-based assessment in wave 1. (b) CAWI W2: web-based interview in wave 2. (c) CATI W3: telephone interview in wave 3. (d) CAWI W4: web-based interview in wave 4.

Table S2.

Logit Regression for Self-Selection by Assessment Mode

	Predictor	Coding	Mod e	*B*	95% CI
Main effects for assessment mode (dummy-coded with WBA as reference category)					
			PBA	-3.10*	(-5.16, -1.04)
			CBA	-0.97	(-3.45, 1.51)
Interactions for selection variable and assessment mode					
Sociodemographics	Gender	0 = male	PBA	-0.17	(-0.54, 0.20)
		1 = female	CBA	-0.17	(-0.53, 0.18)
	Country of birth	0 = other	PBA	-0.16	(-0.67, 0.36)
		1 = Germany	CBA	-0.18	(-0.89, 0.54)
	Mother tongue	0 = other	PBA	0.02	(-0.42, 0.45)
		1 = German	CBA	0.06	(-0.56, 0.68)
	Children in household	0 = no	PBA	-0.29	(-0.94, 0.36)
		1 = yes	CBA	-0.38	(-1.36, 0.61)
	Household size	0 = 1 person	PBA	-0.47	(-1.08, 0.15)
		1 = 2 persons	CBA	-0.47	(-1.27, 0.33)
		0 = 1 person	PBA	-0.36	(-0.97, 0.24)
		1 = 3+ persons	CBA	-0.23	(-1.02, 0.56)
	Year of birth	0 = before 1989	PBA	-0.16	(-0.38, 0.05)
		1 = 1989 or 1990	CBA	0.04	(-0.22, 0.30)
		0 = before 1989	PBA	0.08	(-0.19, 0.34)
		1 = after 1990	CBA	-0.19	(-0.48, 0.11)
	Years of education of mother		PBA	0.02	(-0.02, 0.06)
			CBA	0.04	(-0.01, 0.09)
	Years of education of father		PBA	0.04	(0.00, 0.07)
			CBA	-0.02	(-0.07, 0.02)
Personality	Extraversion		PBA	0.04	(-0.15, 0.23)
			CBA	0.09	(-0.09, 0.27)
	Agreeableness		PBA	0.02	(-0.14, 0.19)
			CBA	0.03	(-0.17, 0.24)
	Conscientiousness		PBA	0.15	(-0.01, 0.31)
			CBA	-0.01	(-0.18, 0.16)
	Neuroticism		PBA	0.08	(-0.07, 0.24)
			CBA	0.07	(-0.11, 0.24)
	Openness		PBA	0.06	(-0.06, 0.18)
			CBA	-0.04	(-0.17, 0.09)
	Self-esteem		PBA	0.00	(-0.03, 0.03)
			CBA	-0.01	(-0.04, 0.03)
Student	Region of Germany	0 = West 1 = East	PBA	0.06	(-0.16, 0.29)
			CBA	0.11	(-0.39, 0.62)
	Field of study	0 = other	PBA	-0.23	(-0.54, 0.08)
		1 = Sciences	CBA	-0.06	(-0.42, 0.31)
		0 = other	PBA	0.24	(-0.03, 0.50)
		1 = Teaching	CBA	-0.06	(-0.36, 0.24)
	University type		PBA	0.01	(-0.30, 0.31)

	0 = General 1 = Applied sciences	CBA	-0.79*	(-1.19, -0.39)
	0 = General 1 = other	PBA	0.13	(-0.61, 0.86)
		CBA	0.06	(-2.45, 2.26)
University admissioncertificate	0 = non-traditional (German) 1 = traditional German	PBA	1.00*	(0.24, 1.72)
		CBA	-0.10	(-1.14, 0.93)
	0 = non-traditional (German) 1 = non-German	PBA	0.87	(-0.21, 1.96)
		CBA	0.06	(-1.51, 1.62)
Average grade in 2nd semester		PBA	-0.11	(-0.33,0.12)
		CBA	-0.17	(-0.33, 0.12)
Mathematical competence		PBA	-0.05	(-0.57, 0.46)
		CBA	-0.04	(-0.49, 0.41)
Enjoy studying		PBA	0.04	(-0.07, 0,15)
		CBA	-0.04	(-0.19, 0.10)
Subjective probability of graduation		PBA	-0.11	(-0.26, 0.04)
		CBA	0.01	(-0.19, 0.20)
Sample Size		17,473		
Pseudo-R^2 / ΔR^2 [(a)]		.25 / .01		

Note. Dependent variable is participation (coded as 1 = participated and 0 = refused). Mode = Dummy-coded with WBA as reference category. *B* = Regression weight for main effect of assessment mode or interaction effects for assessment mode with selection variables. Main effects for selection variables are not presented. R^2 / ΔR^2 = Explained variance / incremental variance explained by moderating effects.

[(a)] Across all imputed data sets the Nagelkerke R^2 values were nearly identical. Thus, the mean R^2 is given.

* $p < .05$

Table S3.

Logit Regressions for Nonresponse Analyses for Students Participating in the Mode-Specific Assessments.

	Variable	Reference category	PBA		CBA		WBA		WBA-switch	
			B	95% CI	*B*	95% CI	*B*	95% CI	*B*	95% CI
Socio-demographics	Gender	Male								
	female		0.09	(-0.19, 0.37)	0.12	(-0.18, 0.42)	0.22*	(0.09, 0.35)	0.29*	(0.14, 0.45)
	Country of birth	not Germany								
	Germany		0.04	(-0.42, 0.50)	-0.10	(-0.78, 0.59)	0.06	(-0.23, 0.34)	0.02	(-0.36, 0.41)
	Mother tongue	non-German								
	German		0.09	(-0.36, 0.55)	0.21	(-0.45, 0.87)	0.07	(-0.17, 0.31)	0.35	(-0.01, 0.70)
	Children in household	no								
	yes		-0.68*	(-1.24, -0.12)	-0.53	(-2.12, 1.06)	-0.46*	(-0.75, -0.18)	-0.10	(-0.52, 0.32)
	Household size	one person								
	two persons		-0.72	(-1.56, 0.11)	-0.63	(-1.98, 0.73)	-0.29	(-0.81, 0.23)	-0.03	(-0.43, 0.33)
	three + persons		-0.83	(-1.67, 0.02)	-0.59	(-1.94, 0.76)	-0.52*	(-1.01, -0.03)	-0.29	(-0.63, 0.05)
	Year of birth	before 1989								
	1989/1990		-0.03	(-0.22, 0.15)	0.15	(-0.09, 0.39)	0.11	(-0.01, 0.24)	0.07	(-0.08, 0.21)
	after 1990		0.16	(-0.06, 0.38)	-0.13	(-0.41, 0.14)	0.05	(-0.09, 0.19)	0.01	(-0.15, 0.17)
	Years of education of mother		0.02	(-0.01, 0.05)	0.04	(-0.01, 0.08)	-0.01	(-0.03, 0.01)	0.01	(-0.02, 0.04)
	Years of education of father		0.03	(-0.01, 0.06)	-0.04	(-0.08, 0.01)	-0.01	(-0.03, 0.02)	-0.02	(-0.04, 0.01)

Table S3. (continued)

	Variable	Reference category	PBA		CBA		WBA		WBA-switch	
			B	95% CI	*B*	95% CI	*B*	95% CI	*B*	95% CI
Personality	Extraversion		-0.10	(-0.24, 0.04)	-0.03	(-0.18, 0.12)	-0.14*	(-0.21, -0.06)	-0.12*	(-0.21, -0.03)
	Agreeableness		0.01	(-0.14, 0.15)	0.02	(-0.17, 0.21)	-0.02	(-0.13, 0.09)	-0.08	(-0.20, 0.05)
	Conscientiousness		0.16*	(0.03, 0.29)	0.01	(-0.15, 0.17)	-0.02	(-0.10, 0.06)	0.05	(-0.06, 0.16)
	Neuroticism		-0.02	(-0.12, 0.09)	-0.04	(-0.20, 0.12)	-0.05	(-0.12, 0.02)	0.00	(-0.09, 0.08)
	Openness		-0.02	(-0.12, 0.09)	-0.11	(-0.24, 0.01)	-0.09*	(-0.16, -0.02)	-0.07	(-0.15, 0.01)
	Self-esteem		0.02*	(-0.01, 0.05)	0.01	(-0.02, 0.04)	0.02*	(0.00, 0.04)	0.00	(-0.02, 0.01)
Student characteristics	Region of Germany	West								
	East		0.03	(-0.15, 0.21)	0.00	(-0.47, 0.47)	0.00	(-0.12, 0.11)	0.36*	(0.20, 0.52)
	Study of natural sciences	no								
	yes		-0.19*	(-0.43, -0.05)	-0.06	(-0.39, 0.27)	0.09	(-0.04, 0.22)	-0.39*	(-0.55, -0.23)
	Study of teacher education	no								
	yes		0.41*	(0.20, 0.63)	0.08	(-0.17, 0.34)	0.16*	(0.04, 0.29)	-0.11	(-0.26, 0.04)
	Public institution	no								
	yes		0.82*	(0.11, 1.53)	--[(f)]	--	0.19	(-0.07, 0.46)	-0.03	(-0.52, 0.47)
	Institution type	general								
	Applied science		-0.06	(-0.33, 0.21)	-0.96*	(-1.33, -0.58)	-0.19*	(-0.33, -0.05)	0.27*	(0.11, 0.43)
	other		0.22	(-0.36, 0.80)	0.62	(-1.92, 3.15)	0.07	(-0.15, 0.29)	0.75*	(0.30, 1.19)
	University admission certificate	non-traditional								
	traditional		1.19*	(0.44, 1.93)	0.10	(-0.93, 1.13)	0.21	(-0.07, 0.49)	0.50*	(0.07, 0.93)
	non-German		0.54	(-0.46, 1.53)	-0.23	(-1.72, 1.26)	-0.33	(-0.85, 0.19)	-0.21	(-1.00, 0.58)
	Average grade on 2nd semester		-0.10	(-0.28, 0.07)	-0.15	(-0.40, 0.10)	0.01	(-0.11, 0.13)	0.02	(-0.10, 0.15)
	Mathematical competence		0.01	(-0.36, 0.38)	0.04	(-0.34, 0.43)	0.04	(-0.06, 0.13)	-0.02	(-0.11, 0.07)
	Enjoy studying		0.05	(-0.05, 0.15)	-0.06	(-0.20, 0.07)	0.03	(-0.03, 0.09)	-0.02	(-0.09, 0.06)
	Probability of graduation		-0.03	(-0.15, 0.10)	0.11	(-0.07, 0.28)	0.07	(-0.01, 0.15)	0.13*	(0.03, 0.22)

Table S3. (continued)

	Variable	Reference category	PBA		CBA		WBA		WBA-switch	
			B	95% CI	*B*	95% CI	*B*	95% CI	*B*	95% CI
Participation in previous waves	PBA W1[a]	no								
	yes		1.29*	(0.74, 1.85)	1.05*	(0.33, 1.77)	0.55*	(0.37, 0.74)	0.26*	(0.06, 0.46)
	CAWI W2[b]	no								
	yes		0.29*	(0.11, 0.48)	0.26*	(-0.01, 0.53)	0.71*	(0.59, 0.82)	0.62*	(0.47, 0.77)
	CATI W3[c]	no								
	yes		0.72*	(0.21, 1.22)	0.69*	(0.21, 1.18)	0.74*	(0.26, 1.21)	0.60*	(0.23, 0.97)
	CAWI W4[d]	no								
	yes		0.81*	(0.63, 0.99)	0.91*	(0.64, 1.19)	1.39*	(1.29, 1.50)	1.05*	(0.91, 1.19)
	Previous mode	CBA								
	PBA		--	--	--	--	--	--	0.18*	(0.05,0.31)
Sample Size			5,371		3,431		8,671		6,804	
Pseudo-R^2 (Nagelkerke)[e]			0.27		0.22		0.31		0.18	

Note. Dependent variable is response (coded as 1 = response and 0 = nonresponse). (a) PBA W1: paper-based assessment in wave 1. (b) CAWI W2: web-based interview in Wave 2. (c) CATI W3: telephone interview in wave 3. (d) CAWI W4: web-based interview in wave 4. (e) Over all imputed data sets the pseudo R^2 values are nearly identical. Thus, here the mean value of the pseudo R^2 values derived using the single imputed data sets is reported. (f) For administrative reasons, CBA was not administered at private universities.

* $p < .05$

Table S4.

Linear Regressions Evaluating Prediction Bias for Full Sample.

	Criterion:	Grade point average		Academic self-concept		Study-related helplessness		Intention to quit	
	Predictor	*B*	95% CI	*B*	95% CI	*B*	95% CI	*B*	95% CI
	Intercept	0.09	(0.00, 0.30)	-0.13*	(-0.21, -0.06)	0.10*	(0.01, 0.28)	0.05	(-0.03, 0.13)
	Main effect of science								
1.	Scientific literacy	-0.08	(-0.14, 0.01)	0.21*	(0.14, 0.27)	0.06*	(-0.34, -0.09)	-0.08*	(-0.16, -0.01)
	Main effects of mode								
2.	CBA	-0.12	(-0.30, 0.06)	0.17*	(0.05, 0.29)	-0.12	(-0.26, 0.02)	0.01	(-0.13, 0.16)
3.	WBA	-0.01	(-0.11, 0.09)	0.12*	(0.03, 0.22)	-0.07	(-0.16, 0.01)	-0.01	(-0.11, 0.08)
4.	WBA-switch	-0.02	(-0.12, 0.09)	0.11*	(0.01, 0.20)	-0.07	(-0.16, 0.02)	0.06	(-0.03, 0.15)
	Moderating effects								
5.	1. x 2.	-0.01	(-0.15, 0.14)	-0.04	(-0.15, 0.07)	-0.07	(-0.19, 0.06)	-0.04	(-0.17, 0.08)
6.	1. x 3.	0.00	(-0.08, 0.08)	-0.10*	(-0.18, -0.02)	-0.02	(-0.10, 0.06)	-0.02	(-0.10, 0.07)
7.	1. x 4.	0.00	(-0.08, 0.09)	-0.09	(-0.18, 0.00)	0.01	(-0.10, 0.12)	0.01	(-0.09, 0.11)
	Covariates								
8.	Sex	-0.33*	(-0.40, -0.26)	0.05	(-0.02, 0.11)	-0.02	(-0.08, 0.05)	-0.06	(-0.13, 0.01)
9.	Teacher education	-0.09*	(-0.17, -0.01)	0.02	(-0.06, 0.09)	0.01	(-0.07, 0.08)	0.00	(-0.07, 0.07)
	R^2 / ΔR^2	.03 / .00		.02 / .00		.01 / .00		.01 / .00	

Note. N = 8,442. Linear regression of outcomes on science literacy, assessment mode (dummy-coded with PBA as reference), respective interactions, and covariates. Sex (-0.5 = male, 0.5 = female) and teacher education (0.5 = other study, -0.5 = teacher education) were effect-coded. Outcomes and scientific literacy were z-standardized. R^2 / ΔR^2 = Explained variance / incremental variance explained by moderating effects. PBA = standardized and supervised paper-based assessment, CBA = standardized and supervised computer-based assessment, WBA = unstandardized and unsupervised web-based assessment with random assignment, WBA-switch = unstandardized and unsupervised web-based assessment with non-random assignment (for PBA / CBA nonresponders).

* $p < .05$
